# Arg^972^ Insulin receptor substrate-1 is associated with decreased serum angiotensin-converting enzyme 2 levels in acute myocardial infarction patients: in vivo and in vitro evidence

**DOI:** 10.1186/1475-2840-12-151

**Published:** 2013-10-17

**Authors:** Wei Liu, Xinmin Zhou, Fenglei Yu, Jianguo Hu, Wen Hu

**Affiliations:** 1 Department of Thoracic and Cardiovascular Surgery, Xiangya Hospital, Central South University, Changsha, Hunan 410008, P.R. China; 2 Department of Thoracic and Cardiovascular Surgery, Second Xiangya Hospital, Central South University, 138 Renmin Road, Changsha, Hunan 410011, P.R. China

**Keywords:** Insulin receptor substrate-1, Gene polymorphism, Angiotensin-converting enzyme 2, Acute myocardial infarction

## Abstract

**Background:**

Activation of the renin-angiotensin system (RAS) plays a critical role in the pathophysiology of myocardial infarction (MI) and the development of heart failure. Both angiotensin-converting enzyme 2 (ACE2) and insulin/insulin receptor substrate-1 (IRS-1) show cardioprotective effects after acute MI. The Arg^972^ IRS-1 polymorphism is associated with diminished activity of insulin. In the present study, we explored the association among Arg^972^ IRS-1, acute MI, and serum levels of ACE2.

**Methods:**

A total of 711 subjects, including 351 subjects with first-time acute MI and 360 subjects without a history of MI were genotyped for Arg^972^ IRS-1 polymorphism. Serum levels of ACE2 and MI severity scores were determined. Primary human cardiomyocytes with overexpression of wild type IRS-1 or Arg^972^ IRS-1 or knockdown of endogenous IRS-1 were exposed to normoxia and hypoxia, and the expression levels of ACE2 were determined.

**Results:**

The serum ACE2 level was significantly increased in acute MI patients compared with that of non-MI controls. Compared with wild type IRS-1 carriers, Arg^972^ IRS-1 carriers exhibited decreased serum ACE2 levels and increased MI severity scores after MI. Our in vitro data demonstrate that impairment of insulin/IRS-1/PI3K signaling by overexpression of Arg^972^-IRS-1, knockdown of endogenous IRS-1, or PI3K inhibitor can abolish hypoxia-induced IRS-1-associated PI3K activity and ACE2 expression in human cardiomyocytes, which suggests a causal relationship between Arg^972^-IRS-1 and decreased serum ACE2 levels in acute MI patients. Our in vitro data also indicate that insulin/IRS-1/PI3K signaling is required for ACE2 expression in cardiomyocytes, and that hypoxia can enhance the induction effect of insulin/IRS-1/PI3K signaling on ACE2 expression in cardiomyocytes.

**Conclusions:**

This study provides the first evidence of crosstalk between insulin/IRS-1/PI3K signaling and RAS after acute MI, thereby adding fresh insights into the pathophysiology and treatment of acute MI.

## Background

Activation of the renin-angiotensin system (RAS) plays a critical role in the pathophysiology of myocardial infarction (MI) and the development of heart failure [1]. RAS blockade with angiotensin-converting enzyme (ACE) inhibitors improves cardiac remodeling and outcomes in both experimental models of MI as well as in humans [1]. ACE metabolizes angiotensin (Ang) I to form Ang II, which is of key importance in the pathophysiology of the RAS in the heart [2]. Recently, ACE2, a new member of the RAS, was found to function as a negative regulator of the Ang system by metabolizing Ang II to a putatively protective peptide Ang-(1–7) with high efficiency [3]. ACE2 is present in the heart, and a reduction in its expression is associated with enhanced cardiac hypertrophy and reduced pumping ability [4,5]. Although ACE2 was initially localized exclusively in cardiac endothelial cells, more recent studies demonstrate ACE2 immunoreactivity in both the endothelial and smooth muscle cells of myocardial vessels as well as in cardiomyocytes [4,5]. After MI, there is significant activation of cardiac ACE2 in rats and humans, which acts to combat the adverse effects of an activated cardiac RAS [4]. Other evidence for a cardioprotective role for ACE2 arises from studies in ACE2-knockout mice where the loss of ACE2 facilitates adverse post-MI ventricular remodeling, and studies showing that ACE2 overexpression in MI rats improves cardiac contractility and remodeling [6,7]. It has been reported that ACE2 serum activity rises during the first week following acute MI and that ACE2 activation may be a compensatory mechanism in advanced heart failure [8].

Insulin has beneficial effects on cardiomyocyte function and survival, including protection against acute ischemic injury [9]. Insulin signaling can promote glucose uptake, glycolytic flux and glucose oxidation, and improve ischemic tolerance of the heart [10-12]. The protective effects of insulin on the heart are mediated via activation of a signaling pathway involving the insulin receptor, insulin receptor substrate-1 (IRS-1), and phosphoinositide-3 kinase (PI3K) [9,13,14]. Previous studies have demonstrated that a common polymorphism in the IRS-1 gene, the Arg^972^ IRS-1 polymorphism, in which a Gly/Arg substitution takes place at codon 972 (Arg^972^), is associated with impaired IRS-1 ability to activate the downstream factor phosphatidylinositol-3 kinase (PI3K), leading to diminished activity of insulin [15,16].

Results from one of our pilot studies suggested that the Arg^972^ IRS-1 polymorphism might be associated with ACE2 activation in MI patients. In the present study, we explored the association among Arg^972^ IRS-1, acute MI, and serum levels of ACE2 in a relatively large sample of acute MI patients, with an aim to identify potential interaction between insulin/IRS-1 signaling and RAS in the pathophysiology of acute MI.

## Methods

### Subjects

Between July 2010 and October 2012, 351 subjects with first-time acute MI (mean age 62.7±15.9 years; range, 27–78 years) were recruited to this study at the Second Xiangya Hospital of Central South University. Inclusion criteria were: (1) patients who had undergone a first attack of ST-elevation myocardial infarction (STEMI); (2) verbally communicable; (3) those who agreed to participate in the study. Subjects without a history of MI (mean age 61.2±13.5 years; range, 25–75 years) were enrolled as controls (n=360). Subjects with congenital heart diseases were excluded. Blood samples were drawn on day 7 post MI from the acute MI subjects, and on day 1 from the controls after they consented to participate in the study. All blood samples were subject to ELISA assays for serum ACE2 levels. This study was approved by the Ethics Committee of the Second Xiangya Hospital, Central South University. Written informed consents were obtained from all participants before the start of the study.

### Genotyping

The Arg^972^ IRS-1 polymorphism was identified by restriction fragment length polymorphism as previously described [17]. Three single nucleotide polymorphisms (SNPs), 1075A/G (rs1978124), 8790A/G (rs2285666) and 16854G/C (rs4646142) were selected as proxies to study ACE2 polymorphisms as previously described [18].

### MI severity score

We used the Predicting Risk of Death in Cardiac Disease Tool (PREDICT) score to create an index of severity as previously described [19]. It was used to provide a simple, long-term admission-day prognostic score for patients hospitalized for MI or unstable angina. Score components include shock (0 to 4 points), clinical history (MI, stroke, angina; 0 to 2 points), age (0 to 3 points), ECG findings (0 to 3 points), congestive heart failure (0 to 3 points), and Charlson Comorbidity Index (0 to 6 points) for a maximum severity score of 21 points [19].

### Plasmids and reagents

A fragment of human genomic DNA containing the entire coding sequence of IRS-1 was cloned and ligated into pcDNA3.1 expression vector (pcDNA-WT-IRS-1) as previously described [20]. The pcDNA-Arg972-IRS-1 expression vector was constructed as previously described [16]. Superfect™ transfection reagent was purchased from Qiagen (Valencia, CA, USA). TRIzol reagent for RNA isolation, and the SYBR Green Master Mix were purchased from Invitrogen (Carlsbad, CA, USA) and PE Applied Biosystems (Foster City, CA, USA), respectively. IRS-1 (sc-29376-V) short hairpin RNA (shRNA) lentiviral particles, control shRNA lentiviral particles-A (sc-108080), and anti-ACE2 (sc-73668) antibody were purchased from Santa Cruz Biotechnology (Santa Cruz, CA, USA). ACE2 (human) ELISA Kit (AG-45A-0022EK-KI01) was purchased from Adipogen (San Diego, CA, USA). SensoLyte® 520 TACE (α-Secretase) activity assay kit (72085) was purchased from AnaSpec (Fremont, CA, USA). Puromycin, LY294002 and all other chemicals of reagent grade were purchased from Sigma (St. Louis, MO, USA).

### Cell culture, lentiviral transduction, transfection of IRS-1 cDNAs, and application of hypoxia treatment

Human adult cardiomyocytes (#6210) and cardiomyocyte medium (#6210) were purchased from ScienCell Research Laboratories (Carlsbad, CA, USA). Lentiviral transduction was performed in human cardiomyocytes. The IRS-1 shRNA lentiviral particles contain expression constructs encoding target-specific 19–25 nt (plus hairpin) shRNA designed to specifically knock down IRS-1 gene expression. The control shRNA lentiviral particles contain a scrambled shRNA sequence that will not lead to degradation of any cellular mRNA, and was used as a negative control for IRS-1 shRNA lentiviral particles. Pools of stable transductants were generated via selection with puromycin (5 μg/mL) by the manufacturer’s protocol (Santa Cruz Biotechnology).

Human cardiomyocytes were stably transfected with empty pcDNA3.1, pcDNA-WT-IRS-1 or pcDNA-Arg972-IRS-1 plasmids using Superfect™ transfection reagent (Qiagen) according to the manufacturer's instructions. Pools of stable transfectants were generated via selection with puromycin (5 μg/ml) by the manufacturer’s protocol. For hypoxia treatment, cells were respectively kept at 1% O_2_ for 24 hours in serum-free medium with or without 1 mg/L insulin [21].

### Real-time quantitative reverse transcription PCR

RNA from human cardiomyocytes were prepared using TRIzol reagent followed by purification with TURBO DNA-free System (Ambion, Austin, TX, USA). The cDNAs were synthesized using SuperScript II reverse transcriptase (Invitrogen). Real-time quantitative PCR was performed on an Abi-Prism 7700 Sequence Detection System (Applied Biosystems), using the fluorescent dye SYBR Green Master Mix (PE Applied Biosystems) as described by the manufacturer. The results were normalized against that of the housekeeping gene glyceraldehyde-3-phosphate dehydrogenase (*GAPDH*) in the same sample. The primers used are as follows: for ACE2, 5′-GCAGCTAAGTATAATGGTTCTCTG -3′ (forward) and 5′-AGTGTTCCACCCCACAAAA -3′ (reverse); for GAPDH, 5′-GTCAGTGGTGGACCTGACCT-3′ (forward) and 5′-TGCTGTAGCCAAATTCGTTG-3′ (reverse). Each experiment was repeated for three times in triplicates.

### Western blot analysis

Human cardiomyocytes were lysed in 250 μ l of 2× SDS loading buffer (62.5 mM TrisHCl, pH 6.8, 2% SDS, 25% glycerol, 0.01% bromphenol blue, 5% 2-mercaptoethanol), and incubated at 95 °C for 10 min. Equal amount of proteins (100 μg) for each sample were separated by 8-15% SDS-polyacrylamide gel and blotted onto a polyvinylidene difluoride microporous membrane (Millipore, Billerica, MA, USA). Membranes were incubated for 1 hour with a 1:1000 dilution of primary antibody, and then washed and revealed using secondary antibodies with horseradish peroxidase conjugate (1:5000, 1 hour). Peroxidase was revealed with a GE Healthcare ECL kit. Proteins were quantified before being loaded onto the gel.

### PI3K Activity assay

IRS-1-associated PI3K activities were determined as previously described [9]. Briefly, 700 μg of total protein was immunoprecipitated with anti-IRS-1 antibody (Santa Cruz Biotechnology Inc.), and kinase activity was detected by the appearance of radiolabeled ^32^P-labeled phosphatidylinositol 3-phosphate ([^32^P]PI-3-P) after TLC as described [22]. Autoradiographic signals were quantitated using NIH Image version 1.63.

### Statistical analysis

Statistical analyses were carried out using SPSS15.0 (IBM, Chicago, IL, USA). All continuous variable values were expressed as Mean±SD. Comparison of means between two independent groups was performed with student t-tests. Comparison of serum ACE2 levels before and after acute MI in the same genotype group was performed with paired t-tests. Comparison of serum ACE2 levels after acute MI between two genotype groups was performed with analysis of covariance using serum ACE2 levels before acute MI as a covariate. Comparisons of means among multiple groups were performed with one-way ANOVA followed by *post hoc* pairwise comparisons using Tukey's tests. Categorical variables were compared with Chi-square tests. The main effect of and interaction among Arg^972^ IRS-1 and MI on serum ACE2 levels were analyzed with ANOVA. The significance level of this study was set at a two-tailed α=0.05.

## Results

In a total of 711 subjects, there were 351 subjects with first-time acute STEMI and 360 controls without a history of MI. Blood samples were collected on day 7 after MI in the acute MI subjects. As shown in Table 1, the acute MI subjects and the controls were comparable in age, BMI, blood lipids (except for low-density lipoprotein cholesterol and triglycerides), blood pressure, and insulin sensitivity. As shown in Table 2, only 2.6%-2.8% of subjects in the control and the acute MI groups were homozygous Arg^972^ IRS-1 (AA) carriers. As the number of Arg^972^ IRS-1 homozygotes was too small to generate any results of adequate statistical power, we combined Arg^972^ IRS-1 homozygotes (AA) and heterozygotes (GA) into one group (GA+AA) to compare with the wild type IRS-1 (GG) group. In addition, as the acute MI subjects and the non-MI controls showed marginally significant difference in the gender profile (*p*=0.08), we performed gender-stratified comparisons between the control subjects and the acute MI subjects to minimize the potential confounding effects of gender. There was no significant difference in the allelic frequency between the acute MI subjects and the non-MI control subjects in total or by gender (Table 2), indicating that the Arg^972^ IRS-1 mutation is not a significant contributor for acute MI.

**Table 1:** Characteristics of study subjects

**Subject characteristics**		**Acute MI (n=351)**	**Control (n=360)**	***p***
Age (years)		62.7±15.9	61.2±13.5	0.18
Age Group (years) n(%)	25-39	22 (6.3)	25(6.9)	
	40-49	47 (13.4)	50 (13.9)	1.00
	50-59	72 (20.5)	73 (20.3)	
	60-69	98 (27.9)	100 (27.8)	
	≥70	112 (31.9)	112(31.1)	0.08
Gender n(%)	Male	225 (64.1)	207 (57.5)	
	Female			
		126 (35.9)	153 (42.5)	
BMI (kg/m^2^)		29.7 ± 9.2	28.6 ± 9.7	0.12
Total Cholesterol (mmol/L)		6.0 ± 3.5	5.6 ± 2.6	0.10
LDL- Cholesterol (mmol/L)		3.9 ± 2.3	3.5 ± 1.8	0.01^a^
HDL- Cholesterol (mmol/L)		1.1 ± 0.7	1.2 ± 0.9	0.10
Triglycerides (mmol/L)		2.4 ± 1.5	2.1 ± 1.2	<0.01^a^
Systolic Blood Pressure (mmHg)		133.5 ± 19.2	131.4 ± 14.5	0.12
Diastolic Blood Pressure (mmHg)		86.2 ± 13.6	84.5 ± 11.5	0.08
Fasting Glucose (mmol/L)		5.4 ± 2.1	5.1 ± 2.4	0.09
Fasting Insulin (μU/mL)		18.3 ± 7.4	17.3 ± 6.9	0.07

Note: For continuous variables, all values were expressed as Mean±SD. Student t tests were performed to compare means between the groups. For categorical variables, all values were expressed as n(%) and comparisons were performed with Chi-square tests. BMI, body mass index; LDL, low-density lipoprotein; HDL, high-density lipoprotein. ^a^*P*<0.05 compared with Control.

**Table 2:** **Association between arg**^**972 **^**irs-1 polymorphism and acute myocardial infarction (mi) in total subjects and by gender**

	**Group**	**GG**	**GA**	**AA**	***p*** *	**G**	**A**	***p*** *
Total	Control	269 (74.7)	81 (22.5)	10 (2.8)	0.86	619 (86.0)	101 (14.0)	0.73
	(n=360)							
	Acute MI	257 (73.2)	85 (24.2)	9 (2.6)		599 (85.3)	103 (14.7)	
	(n=351)							
Male	Control	144 (69.6)	55 (26.6)	8 (3.8)	0.39	343 (82.9)	71 (17.1)	0.72
	(n=207)							
	Acute MI	156 (69.3)	65 (28.9)	4 (1.8)		377 (83.8)	73 (16.2)	
	(n=225)							
Female	Control	125 (81.7)	26 (17.0)	2 (1.3)	0.36	276 (90.2)	30 (9.8)	0.49
	(n=153)							
	Acute MI	101 (80.2)	20 (15.9)	5 (3.9)		222 (88.1)	30 (11.9)	
	(n=126)							

Note: All genotype data are expressed as n(%). *Chi-square *p* values.

As shown in Table 3, the serum ACE2 level was significantly higher in the acute MI subjects than that in the control subjects (*p*<0.05). There was no significant difference in the serum ACE2 level between the GG and the GA+AA genotype groups in the control subjects. However, in the acute MI subjects, the GA+AA genotype group had significantly lower serum ACE2 levels and higher MI severity scores than the GG genotype group (*p*<0.05). There were no significant gender differences in the findings. As shown in Table 4, ANOVA analysis of the whole cohort of subjects (both the control and the STEMI subjects with a discontinuous covariate of acute MI) revealed that acute MI (*p*=0.00), but not the Arg^972^ IRS-1 genotype (*P*=0.19) contributed significantly to the variance of serum ACE2 levels. In addition, the Arg^972^ IRS-1 genotype and acute MI showed significant interaction (*p*=0.00) that contributes to the variance of serum ACE2 levels. The results suggest that after acute MI, Arg^972^ IRS-1 carriers tend to have decreased serum ACE2 levels than wild type IRS-1 carriers. Out of the 351 acute MI subjects, 165 had blood drawn for other studies before the occurrence of MI. We next examined serum ACE2 levels in the 165 subjects before and after MI. As shown in Table 5, the serum ACE2 level in the GA+AA genotype group was similar to that in the GG genotype group before acute MI. However, after the occurrence of acute MI, the GA+AA genotype group had significantly lower serum ACE2 levels than the GG genotype group (*p*<0.05). There were no significant gender differences in the findings. Taken together, the results indicate that the presence of Arg^972^ IRS-1 leads to significantly reduced serum ACE2 levels and more severe MI in acute MI patients independent of gender.

**Table 3:** Serum angiotensin-converting enzyme 2 (ace2) levels and myocardial infarction (mi) scores in control and acute mi subjects

**Subjects**		**Genotype**	**N**	**Serum ACE2 level (ng/mL)**	**MI severity score**
Total	Control	GG	269	6.8±4.5	N/A
		GA+AA	91	5.5±3.3	
		Total	360	6.3±3.9	
	Acute MI	GG	257	15.9±7.8^a^	11.6±6.5
		GA+AA	94	8.1±5.4^b,c^	18.8±4.4^c^
		Total	351	13.7±7.2	13.5±4.9
Male	Control	GG	144	6.6±4.7	N/A
		GA+AA	63	5.2±3.5	
		Total	207	6.2±4.2	
	Acute MI	GG	156	15.7±8.0^a^	11.7±6.7
		GA+AA	69	7.8±5.5^b,c^	19.0±4.6^c^
		Total	225	13.3±7.5	13.8±5.2
Female	Control	GG	125	7.0±4.4	N/A
		GA+AA	28	6.2±3.1	
		Total	153	6.9±3.5	
	Acute MI	GG	101	16.2±7.5^a^	11.4±6.2
		GA+AA	25	8.9±5.2^b,c^	18.2±4.0^c^
		Total	126	14.8±8.0	12.8±4.4

Note: Comparisons of means among genotype groups were performed with one-way ANOVA followed by *post hoc* pairwise comparisons using Tukey's tests in total subjects and by gender. ^a^*p*<0.05 compared with GG in Control subjects; ^b^*p*<0.05 compared with GA+AA in Control subjects; ^c^*p*<0.05 compared with GG in Acute MI subjects.

**Table 4:** **ANOVA analysis of the main effect of and interaction between arg**^**972 **^**irs-1 and acute myocardial infarction (mi) on serum angiotensin-converting enzyme 2 (ace2) levels**

**Dependent variable**	**Acute MI**			**Genotype**			**Acute MI × Genotype**		
	**F**	***p***	**Partial Eta squared**	**F**	***p***	**Partial Eta squared**	**F**	***p***	**Partial Eta squared**
Serum ACE2 Levels	19.22	0.00	0.29	1.69	0.19	0.04	11.98	0.00	0.16

**Table 5:** Serum angiotensin-converting enzyme 2 (ace2) levels before and after acute myocardial infarction (mi)

		**Serum ACE2 level (ng/mL)**	
		**Before acute MI**	**After acute MI**
Total	GG	7.2±4.2	16.4±8.2^a^
(n=165)	(n=116)		
	GA+AA	6.4±4.7	8.5±5.2^b,c^
	(n=49)		
Male	GG	7.0±4.6	16.1±8.5^a^
(n=105)	(n=71)		
	GA+AA	6.1±4.7	8.1±5.7^b,c^
	(n=34)		
Female	GG	7.5±3.7	16.9±7.8^a^
(n=60)	(n=45)		
	GA+AA	7.1±4.6	9.3±4.7^b,c^
	(n=15)		

Note: ^a^*p*<0.05 compared with GG before acute MI by paired t-test; ^b^*p*<0.05 compared with GA+AA before acute MI by paired t-test; ^c^*p*<0.05 compared with GG after acute MI by analysis of covariance using serum ACE2 levels before acute MI as a covariate.

**Figure 1:**
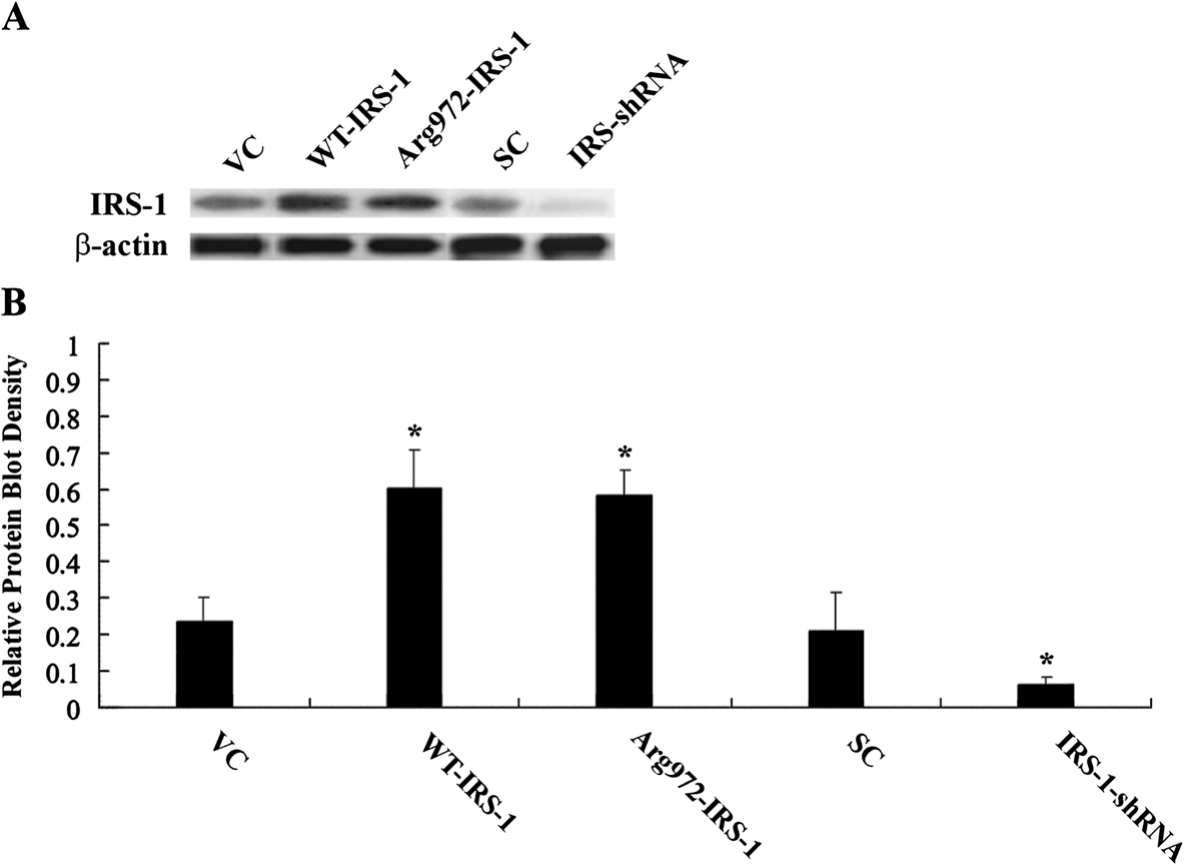
**Western Blot Analysis of IRS-1 Expression in Human Cardiomyocytes. *A*,** human cardiomyocytes were stably transfected with wild type (WT) IRS-1 or Arg^972^ IRS-1, or stably transduced with IRS-1-shRNA. Human cardiomyocytes stably transfected with empty pcDNA3.1 plasmids (VC) or stably transduced with scramble control shRNA (SC) were used as controls. Cell lysates were subject to western blot analysis using an anti-IRS-1 antibody. β-Actin blotting was used as a loading control. ***B*,** protein blots were measured by densitometry. The density of the blots was normalized against that of β-actin to obtain a relative blot density. *p<0.05 compared with VC.

**Figure 2:**
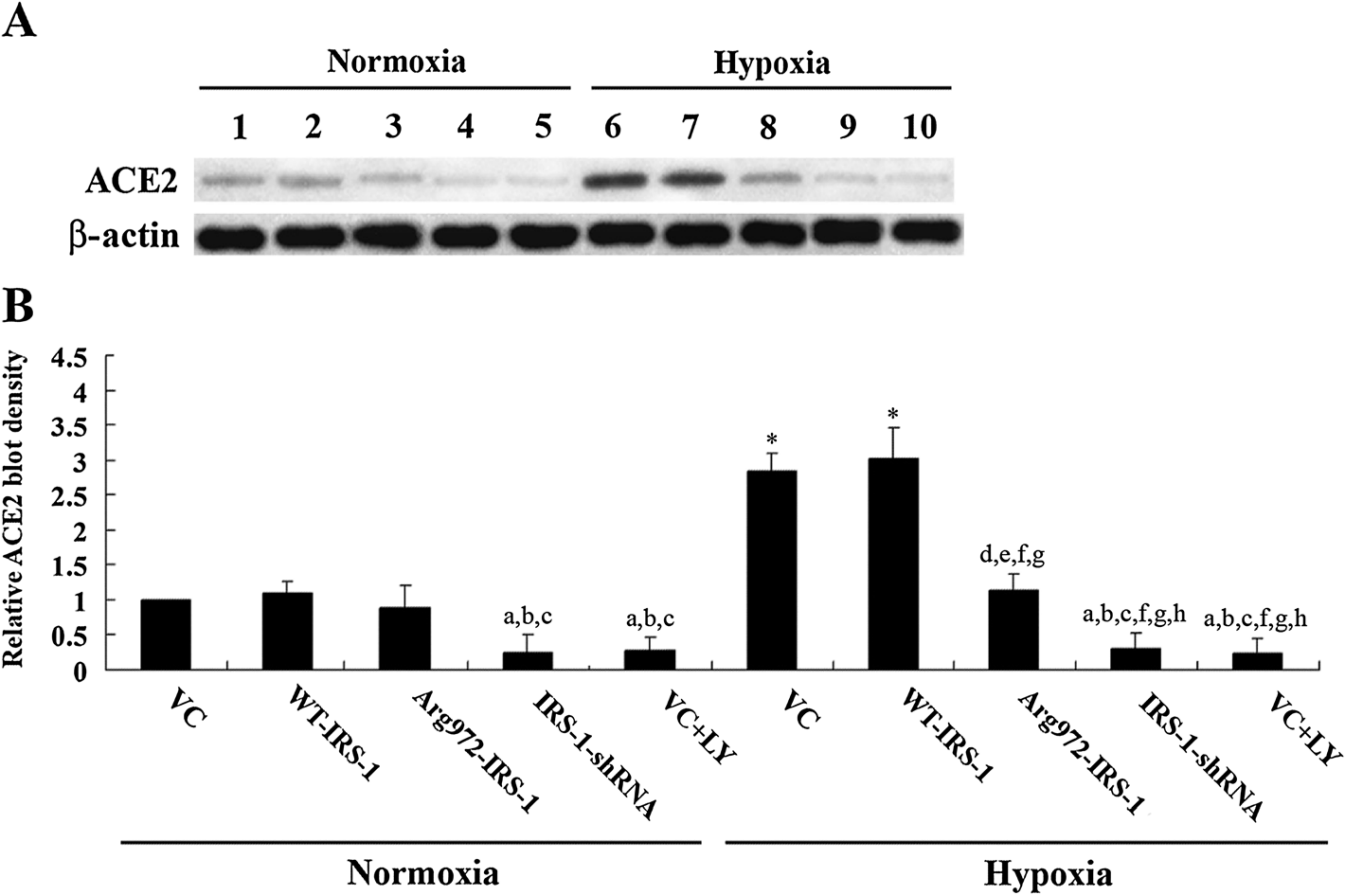
**Western Blot Analysis of Angiotensin-Converting Enzyme 2 (ACE2) Expression in Human Cardiomyocytes under Normoxia and Hypoxia. *A*,** human cardiomyocytes were stably transfected with wild type (WT) IRS-1 or Arg^972^ IRS-1, or stably transduced with IRS-1-shRNA. Then the cells were cultured under normoxia or hypoxia for 24 hours in the presence or absence of LY294002 (LY, 50 μM). Lysates were subject to western blot analysis for ACE2 expression (lanes 1–5, normoxia; lanes 6–10, hypoxia). Lysate from human cardiomyocytes stably transfected with empty pcDNA3.1 plasmids was used as a control (VC). Lanes 1 and 6, VC; lanes 2 and 7, cardiomyocytes stably transfected with WT-IRS-1; lanes 3 and 8, cardiomyocytes stably transfected with Arg972-IRS-1; lanes 4 and 9, cardiomyocytes stably transduced with IRS-1-shRNA; lanes 5 and 10, VC cells treated with LY (50 μM) for 24 hours. β-Actin blotting was used as a loading control. ***B*,** ACE2 and β-actin blots were measured by densitometry. The density of ACE2 blots was normalized against that of β-actin to obtain a relative blot density, which was expressed as fold changes to that of the VC cells under normoxia (designated as 1). *p<0.05 compared with any group in normoxia; ^a^*p*<0.05 compared with VC in normoxia; ^b^*p*<0.05 compared with WT-IRS-1 in normoxia; ^c^*p*<0.05 compared with Arg972-IRS-1 in normoxia; ^d^*p*<0.05 compared with IRS-1-shRNA in normoxia; ^e^*p*<0.05 compared with VC+LY in normoxia; ^f^*p*<0.05 compared with VC in hypoxia; ^g^*p*<0.05 compared with WT-IRS-1 in hypoxia; ^h^*p*<0.05 compared with Arg972-IRS-1 in hypoxia.

**Figure 3:**
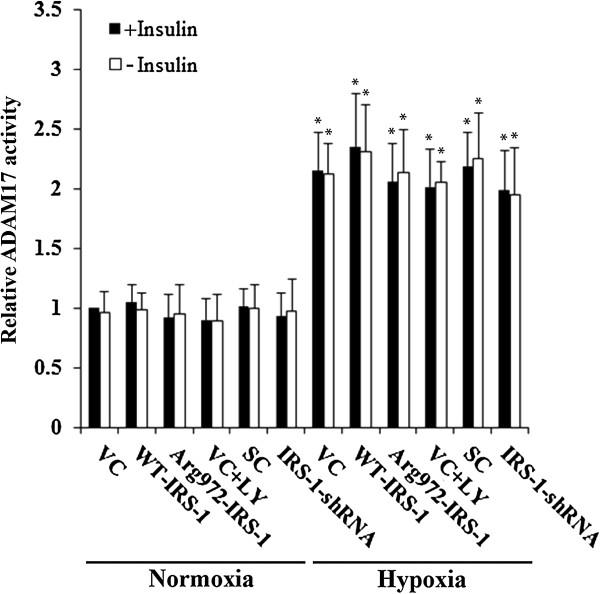
**ADAM17 Shedding Activity in Human Cardiomyocytes under Normoxia and Hypoxia with or without Insulin.** Human cardiomyocytes were stably transfected with wild type (WT) IRS-1 or Arg^972^ IRS-1, or stably transduced with IRS-1-shRNA. Then the cells were cultured under normoxia or hypoxia for 24 hours in the presence or absence of LY294002 (LY, 50 μM) or insulin (1 mg/L). Human cardiomyocytes stably transfected with empty pcDNA3.1 plasmids (VC) or stably transduced with scramble control shRNA (SC) were used as controls. The ADAM17 shedding activity was determined by ELISA and expressed as fold changes to that of the VC cells under normoxia+insulin (1 mg/L) (designated as 1). *p<0.05 compared with any group in normoxia.

To explore the potential causal relationship between Arg^972^ IRS-1 and serum ACE2 levels in acute MI subjects, we performed in vitro experiments using human cardiomyocytes treated with hypoxia. Expression vectors for wild type IRS-1 and Arg^972^ IRS-1 were stably transfected into cardiomyocytes. Endogenous IRS-1 was stably knocked down by shRNA. As shown in Figure 1, stably transfected cells showed marked increase in the protein detected by an anti-IRS-1 antibody, indicating that wild type IRS-1 or Arg^972^ IRS-1 was overexpressed in the transfected cardiomyocytes. Compared with cells stably transduced with scramble control shRNA, cells stably transduced with IRS-1 shRNA showed over 80% knockdown of endogenous IRS-1 (Figure 1). Real-time quantitative RT-PCR showed that in the absence of insulin, the ACE2 mRNA level remained at a basal level in all experimental groups under either normoxia or hypoxia (Table 6). In the presence of insulin (1 mg/L), the ACE2 mRNA level was significantly elevated in the controls. In normoxia, while overexpression of wild type or Arg^972^ IRS-1 did not significantly change the ACE2 mRNA level, both IRS-1 shRNA and PI3K inhibitor LY294002 abolished insulin-induced elevation of the ACE2 mRNA level (Table 6). Cardiomyocytes were then exposed to hypoxia to simulate oxygen deprivation during MI. After 24 hours of culture under hypoxia plus insulin (1 mg/L), the control cells showed significantly elevated ACE2 mRNA levels compared to the controls under normoxia+insulin (Table 6). Overexpression of Arg^972^ IRS-1 markedly decreased the ACE2 mRNA level in cardiomyocytes under hypoxia (Table 6). Both IRS-1 shRNA and LY294002 abolished the induction of ACE2 mRNA by insulin plus hypoxia (Table 6). The results were confirmed at the ACE2 protein level by western blot analyses, which also showed that ACE2 was expressed at a constitutively low level in cardiomyocytes under normoxia (Figure 2). ELISA showed that secreted/soluble ACE2 levels in the culture media of cardiomyocytes followed the same data trend (Table 7). The findings indicate that insulin/IRS-1/PI3K signaling is required for ACE2 expression in cardiomyocytes, and that hypoxia can markedly enhance the induction effect of insulin/IRS-1/PI3K signaling on ACE2 expression in cardiomyocytes. As ACE2 reportedly is shed by sheddase ADAM17 to produce soluble ACE2 [23], and ADAM17 expression reportedly is regulated by hypoxia [24], we next examined ADAM17 shedding activities in human cardiomyocytes under the same experimental conditions as in Table 7. As shown in Figure 3, the ADAM17 shedding activity was significantly up-regulated by hypoxia in cardiomyocytes. However, overexpression or knockdown of IRS-1, overexpression of Arg^972^ IRS-1, or inhibition of PI3K showed no significant effects on the ADAM17 activity in cardiomyocytes with or without insulin, indicating that insulin signaling had no significant effect on the ADAM17 shedding activity in cardiomyocytes. Therefore, the effects of Arg^972^ IRS-1 on ACE2 expression in cardiomyocytes did not involve a shedding mechanism. In addition, we found no association between Arg^972^-IRS-1 variants and the ACE2 1075AA, 16854GG or 8790AA genotypes in female acute MI patients, or the ACE2 1075A/G-8790A/G-16854G/C haplotype GGC in male acute MI patients (data not shown).

**Table 6:** Relative mrna levels of angiotensin-converting enzyme 2 (ace2) in human cardiomyocytes under normoxia and hypoxia with or without insulin treatment

**Group**	**Normoxia**		**Hypoxia**	
	**+Insulin**	**-Insulin**	**+Insulin**	**-Insulin**
VC	0.32±0.11	0.07±0.04	0.79±0.15*	0.08±0.05
WT-IRS-1	0.34±0.08	0.09±0.06	0.82±0.13*	0.10±0.05
Arg972-IRS-1	0.27±0.10	0.05±0.03	0.38±0.08^a,b,c^	0.06±0.03
VC+LY	0.06±0.04	0.04±0.02	0.09±0.05^a,b,c,d^	0.04±0.02
SC	0.30±0.12	0.06±0.03	0.80±0.13*	0.07±0.04
IRS-1-shRNA	0.08±0.05	0.04±0.02	0.11±0.06^a,b,c,d^	0.06±0.03

Note: Human cardiomyocytes were stably transfected with wild type (WT) IRS-1 or Arg^972^ IRS-1, or stably transduced with IRS-1-shRNA. Then the cells were cultured under normoxia or hypoxia for 24 hours in the presence or absence of LY294002 (LY, 50 μM) or insulin (1 mg/L). Human cardiomyocytes stably transfected with empty pcDNA3.1 plasmids was used as a control (VC). *p<0.05 compared with any group under normoxia; ^a^*p*<0.05 compared with VC or SC under hypoxia+insulin (1 mg/L); ^b^*p*<0.05 compared with WT-IRS-1 under hypoxia+insulin (1 mg/L); ^c^*p*<0.05 compared with Arg972-IRS-1 under normoxia+insulin (1 mg/L); ^d^*p*<0.05 compared with Arg972-IRS-1 under hypoxia+insulin (1 mg/L).

**Table 7:** Secreted/soluble angiotensin-converting enzyme 2 (ace2) levels in culture media of human cardiomyocytes under normoxia and hypoxia with or without insulin treatment

**Group**	**Normoxia**		**Hypoxia**	
	**+Insulin**	**-Insulin**	**+Insulin**	**-Insulin**
VC	4.2±1.4	0.8±0.4	19.2±5.3*	1.0±0.5
WT-IRS-1	4.5±1.2	0.9±0.6	20.7±5.5*	1.3±0.7
Arg972-IRS-1	3.8±1.4	0.6±0.4	9.3±3.2*^,a,b^	0.8±0.3
VC+LY	0.5±0.4	0.5±0.3	0.5±0.3^a,b,c^	0.5±0.4
SC	4.0±1.1	0.8±0.3	20.3±5.6*	1.1±0.6
IRS-1-shRNA	0.6±0.3	0.5±0.4	0.7±0.5^a,b,c^	0.6±0.4

Note: Human cardiomyocytes were stably transfected with wild type (WT) IRS-1 or Arg^972^ IRS-1, or stably transduced with IRS-1-shRNA. Then the cells were cultured under normoxia or hypoxia for 24 hours in the presence or absence of LY294002 (LY, 50 μM) or insulin (1 mg/L). Human cardiomyocytes stably transfected with empty pcDNA3.1 plasmids was used as a control (VC). Secreted/soluble ACE2 levels (ng/mL) in the cell culture media were determined by ELISA. *p<0.05 compared with any group under normoxia; ^a^*p*<0.05 compared with VC or SC under hypoxia+insulin (1 mg/L); ^b^*p*<0.05 compared with WT-IRS-1 under hypoxia+insulin (1 mg/L); ^c^*p*<0.05 compared with Arg972-IRS-1 under hypoxia+insulin (1 mg/L).

**Figure 4:**
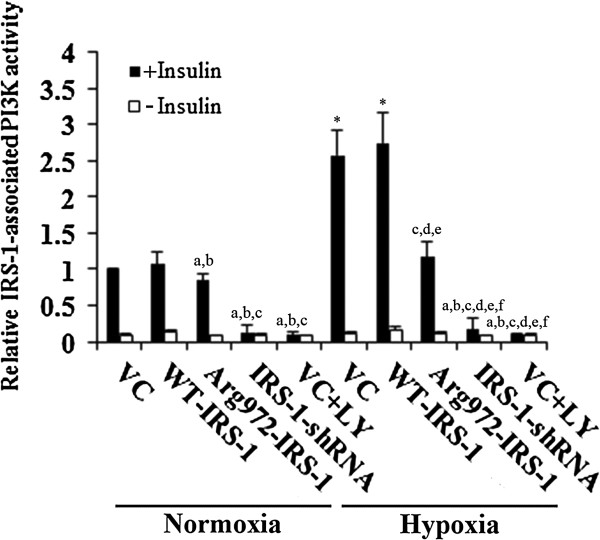
**IRS-1-Associated Phosphatidylinositol-3 Kinase (PI3K) Activity in Human Cardiomyocytes under Normoxia and Hypoxia with or without Insulin.** Human cardiomyocytes were stably transfected with wild type (WT) IRS-1 or Arg^972^ IRS-1, or stably transduced with IRS-1-shRNA. Then the cells were cultured under normoxia or hypoxia for 24 hours in the presence or absence of LY294002 (LY, 50 μM) or insulin (1 mg/L). Human cardiomyocytes stably transfected with empty pcDNA3.1 plasmids was used as a control (VC). IRS-1-associated PI3K activities were measured and were expressed as fold changes to that of the VC cells treated with normoxia+insulin (1 mg/L) (designated as 1). *p<0.05 compared with any group in normoxia; ^a^*p*<0.05 compared with VC in normoxia; ^b^*p*<0.05 compared with WT-IRS-1 in normoxia; ^c^*p*<0.05 compared with Arg972-IRS-1 in normoxia; ^d^*p*<0.05 compared with VC in hypoxia; ^e^*p*<0.05 compared with WT-IRS-1-shRNA in hypoxia; ^f^*p*<0.05 compared with Arg972-IRS-1 in hypoxia.

As shown in Figure 4, in the absence of insulin, the IRS-1-associated PI3K activity remained at a basal level in all experimental groups under either normoxia or hypoxia. In the presence of insulin (1 mg/L), the IRS-1-associated PI3K activity was significantly elevated in the controls. In normoxia, overexpression of Arg^972^ IRS-1 slightly decreased the IRS-1-associated PI3K activity, and both IRS-1 shRNA and LY294002 abolished insulin-induced elevation of the IRS-1-associated PI3K activity. In the presence of hypoxia plus insulin (1 mg/L), the control cells showed markedly elevated the IRS-1-associated PI3K activity compared to the controls under normoxia+insulin. Overexpression of Arg^972^ IRS-1 markedly decreased the IRS-1-associated PI3K activity in cardiomyocytes under hypoxia. Both IRS-1 shRNA and LY294002 abolished induction of the IRS-1-associated PI3K activity by insulin plus hypoxia. The results indicate that hypoxia can significantly enhance the IRS-1-associated PI3K activity (or, in other words, insulin/IRS-1/PI3K signaling) in cardiomyocytes.

## Discussion

In the present study, we found that Arg^972^ IRS-1 was associated with decreased serum ACE2 levels in acute MI patients. In vitro data revealed that in the presence of insulin, Arg^972^ IRS-1 inhibits ACE2 expression in human cardiomyocytes under hypoxia.

A previous study showed that serum ACE2 activity significantly increased from baseline to 7 days in STEMI patients [8]. On day 7, a significant direct correlation was observed between ACE2 activity and infarct size and left ventricular function index [8]. Thus, in the present study, we measured serum ACE2 levels on day 7 after acute MI. Our preliminary studies with non-STEMI subjects showed inconsistent serum ACE2 levels. Therefore, only STEMI subjects were enrolled in the present study.

ACE2 is expressed in the heart and reportedly plays a protective role during MI [1,4,5,9]. Increasing evidence show that the expression of cardiac ACE2 is increased after MI, which acts to combat the adverse effects of an activated cardiac RAS and therefore may be a compensatory mechanism in MI [1,4,8]. In agreement with this, in subjects who had blood samples collected before and after MI, we observed a significant increase in the ACE2 serum level after MI. Arg^972^-IRS-1 carriers had significantly lower serum ACE2 levels and higher MI severity scores compared with wild type-IRS-1 carriers after MI, confirming the cardioprotective role of increased ACE2 levels after MI. The findings also suggest an important role for insulin/IRS-1 signaling in the increased ACE2 expression after MI, since Arg^972^-IRS-1 is associated with impaired IRS-1 ability to activate PI3K, which leads to diminished activity of insulin [15,16]. We demonstrated in vitro that a shedding mechanism was not involved in the effects of Arg^972^ IRS-1 on ACE2 expression in cardiomyocytes, suggesting that insulin/IRS-1/PI3K signaling directly targets ACE2 expression.

Myocardial hypoxia is a major factor in the pathophysiology of MI and is thought to be a prime determinant of the progression to heart failure [25]. Thus, in subsequent in vitro experiments to investigate the potential causal relationship between Arg^972^ IRS-1 and ACE2 expression, we exposed primary human cardiomyocytes to hypoxia. In agreement with the in vivo data, ACE2 expression at both the mRNA and the protein/soluble protein levels was increased under hypoxia compared with that under normoxia, which was abolished by overexpression of Arg^972^ IRS-1, knock down of endogenous IRS-1, or PI3K inhibitor.

Insulin signaling can improve ischemic tolerance of the heart via activation of a signaling pathway involving the insulin receptor, insulin receptor substrate-1 (IRS-1), and PI3K [9-14,26]. Our in vitro data indicate that hypoxia can significantly enhance the IRS-1-associated PI3K activity and thereby insulin/IRS-1/PI3K signaling in cardiomyocytes. Our western blot analysis results showed that ACE2 was expressed at a constitutively low level in cardiomyocytes in normoxia. This may explain why overexpression of Arg^972^ IRS-1 had no significant effect on ACE2 expression in cardiomyocytes in normoxia, where a small amount of endogenous wild type IRS-1 in the cells may well suffice for basal level of insulin/IRS-1/PI3K signaling and ACE2 expression despite the competition of Arg^972^ IRS-1. When the IRS-1-associated PI3K activity and the need for wild type IRS-1 were significantly increased by hypoxia in cardiomyocytes, however, the competition of overexpressed Arg^972^ IRS-1 would manifest significant inhibitory effect on insulin/IRS-1/PI3K signaling and thereby ACE2 expression. This may explain for why only Arg^972^ IRS-1 carriers with acute MI showed decreased serum ACE2 levels.

Recent discoveries on the influence of local tissue RAS in the skeletal muscle, heart, vasculature, adipocytes, and pancreas have led to an improved understanding of how activated tissue RAS influences the development of insulin resistance and diabetes in humans [27]. Our study suggests that insulin/IRS-1/PI3K signaling is involved in regulating local RAS in the heart, which may be a novel topic in both the fields of cardiology and diabetology.

There were several limitations of the present study: (1) The increased serum ACE2 levels in acute MI subjects could be from multiple sources such as vascular smooth muscle cells, endothelial cells, and cardiomyocytes. We only used cardiomyocytes in vitro to identify the causal relationship between Arg^972^ IRS-1 and serum ACE2 levels in acute MI subjects. The increased level of secreted/soluble ACE2 in the culture media of cardiomyocytes under hypoxia did suggest that cardiomyocyte-secreted ACE2 could at least partially account for the increased serum ACE2 levels in acute MI patients. However, it would be interesting in future studies to explore the effects of insulin/IRS-1 signaling on ACE2 expression in other cell types. (2) There are many other pathophysiological factors involved in acute MI besides hypoxia. We only explored the effect of insulin/IRS-1 signaling on ACE2 expression under hypoxia and normoxia. It would be interesting to investigate how insulin/IRS-1 signaling regulates ACE2 expression under the influence of other conditions involved in acute MI (e.g. increased cardiomyocyte apoptosis and inflammatory responses, etc.). Nevertheless, based on in vivo and in vitro data, we have shown a potential causal relationship between Arg^972^ IRS-1 and serum ACE2 levels in acute MI patients. Our findings suggest that insulin/IRS-1/PI3K signaling exerts cardioprotective effects after acute MI at least partially through increased ACE2 expression; in agreement with this, Arg^972^ IRS-1 impairs insulin/IRS-1 signaling and results in decreased ACE2 expression after acute MI, which leads to more severe MI. Thus, insulin/IRS-1/PI3K signaling and ACE2 could be potential new targets for acute MI therapy. In addition, our study suggest that special attention should be paid to acute MI patients carrying Arg^972^ IRS-1, for they tend to have more severe MI and poorer prognosis than those carrying wild type IRS-1.

In conclusion, our in vivo data indicate that Arg^972^-IRS-1 is associated with decreased serum ACE2 levels in acute MI patients. Our in vitro data demonstrate that impairment of insulin/IRS-1/PI3K signaling by overexpression of Arg^972^-IRS-1, knockdown of endogenous IRS-1, or PI3K inhibitor can abolish hypoxia-induced IRS-1-associated PI3K activity and ACE2 expression in human cardiomyocytes, which suggests a causal relationship between Arg^972^-IRS-1 and decreased serum ACE2 levels in acute MI patients. Our in vitro data also indicate that insulin/IRS-1/PI3K signaling is required for ACE2 expression in cardiomyocytes, and that hypoxia can markedly enhance the induction effect of insulin/IRS-1/PI3K signaling on ACE2 expression in cardiomyocytes. This study provides the first evidence of crosstalk between insulin/IRS-1/PI3K signaling and RAS after acute MI, thereby adding fresh insights into the pathophysiology and treatment of acute MI.

## Competing interests

The authors declare that they have no competing interests.

## Authors’ contributions

WL participated in study design, collected data, carried out data analysis, and drafted the manuscript. XZ and FY participated in study design, carried out data analysis, and performed data check and proofreading. JH participated in data collection, carried out data analysis, and performed data check and proofreading. WH participated in study design and data analysis, drafted the manuscript, and performed data check and proofreading. All authors have read and approved the final manuscript.
